# Adiposity, Telomere Length, and Telomere Attrition in Midlife: the 1946 British Birth Cohort

**DOI:** 10.1093/gerona/glx151

**Published:** 2017-08-09

**Authors:** Wahyu Wulaningsih, Diana Kuh, Andrew Wong, Rebecca Hardy

**Affiliations:** MRC Unit for Lifelong Health and Ageing at UCL, London, UK

**Keywords:** Obesity, Life course, Telomere length, Overweight, Cohort

## Abstract

**Background:**

Obesity has been linked with shorter telomere length, both of which have been implicated in ageing, but the impact of early life adiposity on telomere length is unclear.

**Methods:**

We included 2,479 participants from the MRC National Survey of Health and Development with measurements of body mass index, waist and hip circumference, and leukocyte telomere length (LTL) at age 53, of whom 1,000 had second measurements at ages 60–64. Relative LTL was measured with rt-PCR. Linear regression was performed to investigate associations between adiposity and LTL. Body mass index from childhood through adulthood was used to assess adiposity across the life course.

**Results:**

We found no cross-sectional associations between adiposity measures and LTL at ages 53 or 60–64. Longitudinally, each unit gain in waist circumference weakly corresponded with a 0.06% (95% CI: −1.31 to 0.10) LTL decrease annually, with association approaching statistical significance (*p* = 0.09). Being overweight at ages 6 and 15 corresponded to a nonsignificant shorter LTL at age 53 and they were associated with 2.06% (95% CI: 0.05–4.08%) and 4.26% (1.98–6.54%) less LTL attrition in midlife, respectively, compared to those who were not overweight.

**Conclusion:**

There is a weak indication that greater telomere loss was seen with greater concurrent body mass index gain. Adolescent overweight corresponded to shorter telomeres in midlife, albeit weakly, and with less subsequent attrition. Our findings point toward potential pathways which may link adiposity and ageing outcomes.

Overweight and obesity, indicated by high body mass index (BMI), accounted for 4% of global disability-adjusted life years (DALYs) in 2010 (1). There is an evidence that early-life obesity and its changes through adulthood are associated with indicators of healthy ageing in later life such as cardiovascular (2,3) and cognitive functions (4), but its underlying mechanism remains unclear.

Telomeres are DNA–protein complexes at the ends of chromosome which maintain chromosomal stability (5). Telomere shortens as an individual ages and therefore telomere attrition is considered a hallmark of ageing (6). There is evidence that shorter telomeres are linked with increased risk of death, as reported in a recent meta-analysis of two cohorts in America and Europe which included adults aged 43–75 (7). It is also suggested that telomere shortening is involved in the development of age-related diseases including cardiovascular disease and cancer (8), both of which are linked with obesity. A cross-sectional association between high BMI or obesity and shorter telomere length has been reported (9). However, a recent meta-analysis including over 8,000 individuals showed no difference in telomere length between obese individuals and those with normal weight (10); nevertheless, high heterogeneity was reported (*I*^2^ = 99%). Apart from variability arising from different assay or DNA extraction methods (11), within-person variability in this association may need to be teased out since most studies included only had a single measurement of BMI and telomere length (10). Among the few studies investigating repeated measurements of adiposity and telomere length in adult life, conflicting results have been shown (12–15). Little is known regarding the impact of adiposity changes over the life course on telomere length in later life.

We investigated the association between adiposity dynamics and leukocyte telomere length (LTL) measured at ages 53 and 60–64 years among participants of the Medical Research Council (MRC) National Survey of Health and Development (NSHD, also known as the 1946 British Birth Cohort). We evaluated whether change in BMI, waist, or hip circumference was associated with concurrent LTL change between ages 53 and 60–64 years. Furthermore, the repeated recordings of BMI obtained over the life course of NSHD participants allowed investigations of overweight from age 2 onwards in relation to telomere length and telomere attrition.

## Methods

### Study Population

The MRC NSHD has been described in detail elsewhere. This cohort is based on a nationally representative sample of 5,362 births out of all the single births to married mothers that occurred in 1 week in March 1946 in England, Scotland, and Wales. The cohort has been followed up 24 times and the present study used data collected up to 60–64 years when telomere length was last measured (16). At age 60–64, study members still alive and with a known current address in England, Scotland, or Wales were invited for an assessment at one of six clinical research facilities (CRF) or to be visited by a research nurse at home. Invitations were not sent to those who had died (*N* = 778), who were living abroad (*N* = 570), had previously withdrawn from the study (*N* = 594), or had been lost to follow-up (*N* = 564). Of the 2,856 invited participants, 2,229 (78%) were assessed: 1,690 (59%) attended a CRF and the remaining 539 were visited at home. The participating sample remains broadly representative of native born British men and women of the same age.

Ethical approval was obtained from the Greater Manchester Local Research Ethics Committee and the Scotland Research Ethics Committee. Written informed consent was obtained from the study member for each component of each data collection.

### Adiposity Assessment in Midlife

Weight (kg), height (m), waist, and hip circumferences (cm) were measured by trained nurses in a standardized manner at ages 53 and 60–64 years. BMI (kg/m^2^) was calculated from weight and height. We divided BMI into four categories (<18.5, 18.5–25, 25–30, and ≥30 kg/m^2^) according to the National Health Institute (NIH) guidelines and dichotomously into nonoverweight and overweight using 25 kg/m^2^ as a cut-off point. Abdominal obesity was defined as waist circumference >102 cm for men and >88 cm for women. We calculated annual changes in each adiposity measure between ages 53 and 60–64 using the interval between the two measurements.

### BMI at Earlier Ages

Body mass index during childhood through adulthood was calculated from measured height and weight at ages 2, 4, 6, 7, 11, 15, 36, and 43, and from self-reported height and weight at ages 20 and 26. Prior to age 20, overweight status was dichotomized using cut-offs derived from centile curves corresponding to BMI of 25 kg/m^2^ at age 18 in six nationally representative surveys (17).

### Telomere Length Measurement

DNA was extracted from frozen EDTA blood samples using Puregene DNA isolation kits (Flowgen, Leicestershire, UK) (18). Leukocyte telomere length was measured as a ratio (T/S ratio) to standard reference DNA in the same laboratory according to a previously validated real-time polymerase chain reaction (rt-PCR) technique in a blinded fashion (19). Measurements were performed in quadruplicate on an Applied Biosystems 7900HT Fast Real Time PCR system with 384-well plate capacity. The intra-assay coefficient of variation was 2.7% while the inter-assay coefficient of variation was 5.1%. Four internal control DNA samples of known telomere length were used to normalize assays in different runs. LTL measurements were available in 2,479 individuals at 53 years and 1,004 individuals at 60–64 years. For participants with two repeated measurements, we calculated telomere attrition as the annual percentage change in LTL (ΔLTL) as follows: [(LTL_60–64_ – LTL_53_) × 100/ LTL_53_)]/interval (years) between the two measurements.

### Covariates

We used prospective information on socioeconomic position as a potential confounder since it is known to be associated with BMI and with LTL (20). Childhood and adult socioeconomic position (SEP) were based on father’s occupation at age 4 and own occupational class at age 53 years. To categorize SEP, the Registrar General’s classification was used to dichotomize individuals into nonmanual and manual workers. Wherever possible, missing values were imputed from adjacent ages (33 values from age 11 and 14 values from age 15 for childhood SEP; 107 values from age 36; and 107 values from age 43 for adult SEP). Diagnosis of diabetes, coronary heart disease and cancer were self-reported at ages 53 and 60–64.

### Statistical Analysis

Regression models were used to investigate associations of continuous and categorical BMI and overweight status with LTL at ages 53 (*N* = 2,479) and 60–64 (*N* = 1,004) years. LTL and T/S ratio were not normally distributed and was logarithmically transformed and multiplied by 100 (21). Models were subsequently adjusted for sex and for childhood and adult SEP, and then additionally for history of diabetes, coronary heart disease, and cancer. All analyses were repeated replacing BMI by waist and then hip circumference. As some participants had extreme values of LTL, we performed a sensitivity analysis by excluding LTL values below the 5th percentile and above 95th percentile.

Regression models were then used to investigate the association of annual changes in LTL (%) with annual changes in BMI, waist circumference, or hip circumference among those with two repeated measurements (*N* = 1,000). Since weight change explains the majority of change in adult BMI, we also performed a similar analysis using weight instead of BMI. Analyses were conditioned on sex, and childhood and adult SEP, and then additionally adjusted for the baseline adiposity measures at age 53.

To investigate any impact of BMI at earlier ages, regression models were fitted with LTL at age 53 and annual change in LTL between ages 53 and 60–64 as outcomes including using BMI at ages 2, 4, 6, 7, 11, 15, 20, 26, 36, and 43 years separately. Models were adjusted for sex, childhood SEP, adult SEP, and BMI at age 53. Analyses were repeated using past BMI-based overweight status.

All analyses were performed with STATA version 14 (STATA Corp., College Station, TX).

## Results

A total of 2,479 participants had available information on BMI and LTL at age 53, 1,004 had both measurements at age 60–64 and 1,000 at both ages. Characteristics of study participants are presented in Table 1. The mean interval [± standard deviation (*SD*)] between measurements for the subgroup with two measurements was 10.1 ± 1.1 years. Shorter LTL at age 53 was correlated with LTL shortening between age 53 and 60–64.

**Table 1.** Characteristics of Study Participants at Age 53 and 60–64 Years with LTL Measurements

	At 53 Y (*N* = 2,479)		At 60–64 Y (*N* = 1,004)	
	Men	Women	Men	Women
Manual adult socioeconomic status, *N* (%)	484 (38.88)	358 (29.01)	146 (30.74)	130 (24.57)
Manual childhood socioeconomic status, *N* (%)	703 (56.47)	706 (57.21)	257 (54.11)	287 (54.25)
Cancer, *N* (%)	30 (2.41)	76 (6.16)	31 (6.53)	66 (12.48)
Diabetes, *N* (%)	35 (2.81)	35 (2.84)	34 (7.11)	26 (4.91)
Chronic heart disease, *N* (%)	61 (4.90)	31 (2.51)	45 (9.76)	13 (2.51)
BMI (kg/m^2^)	27.39 (3.90)	27.33 (5.31)	27.69 (4.01)	28.05 (5.27)
Waist circumference (cm)	97.67 (10.45)	85.52 (12.49)	100.37 (10.69)	92.44 (12.92)
Hip circumference (cm)	104.20 (6.85)	108.10 (10.53)	104.17 (7.29)	107.00 (10.81)
LTL (T/S ratio)	1.64 (0.93)	1.45 (0.90)	1.09 (0.40)	1.15 (0.40)

*Note*: Data are presented in mean (*SE*), unless otherwise indicated. LTL = leukocyte telomere length.

### Cross-Sectional Associations Between Adiposity and Telomere Length at Ages 53 and 60–64

No cross-sectional associations were seen between BMI, waist circumference or hip circumference and LTL at ages 53 or 60–64 (Table 2, unadjusted models). Similarly, there were no associations when categories of BMI or abdominal obesity were used (Table 2). Associations between adiposity measures, overweight status, and LTL were similar to the unadjusted models after adjustment for sex, and childhood and adult SEP (Table 2), although a weak inverse trend between adiposity and LTL was indicated. Adjustment for history of diabetes, coronary heart disease, and cancer or excluding extreme LTL values did not alter any of these null findings (results not shown).

**Table 2.** Associations Between BMI, Overweight, and LTL at Ages 53 and 60–64

		Unadjusted Model		Multivariable 1*		Multivariable 2^†^	
	*N* (%)	Percent Difference	95% CI	Percent Difference	95% CI	Percent Difference	95% CI
At 53 years							
BMI, kg/m^2^							
Continuous		0.03	−0.51 to 0.45	−0.04	−0.51 to 0.44	−0.07	−0.55 to 0.41
<18.5	6 (0.24)	−2.96	−48.72 to 42.80	−4.19	−49.54 to 41.17	−4.37	−49.75 to 41.00
18.5–25	803 (32.39)	Reference		Reference		Reference	
25–30	1,087 (43.85)	0.48	−4.71 to 5.68	−1.89	−7.08 to 3.30	−2.11	−7.34 to 3.11
≥30	583 (23.52)	2.09	−3.98 to 8.16	1.22	−4.80 to 7.24	0.85	−5.24 to 6.94
Nonoverweight	809 (32.63)	Reference		Reference		Reference	
Overweight or obese	1,670 (67.37)	1.07	−3.71 to 5.85	−0.74	−5.52 to 4.02	−1.04	−5.86 to 3.78
Waist circumference (cm)		0.27	0.09 to 0.44	−0.0007	−0.20 to 0.19	−0.02	−0.28 to 0.18
Hip circumference (cm)		−0.07	−0.33 to 0.17	0.004	−0.24 to 0.26	−0.002	−0.25 to 0.25
Nonabdominally obese	1,652 (66.64)	Reference		Reference		Reference	
Abdominally obese	827 (33.36)	0.64	−4.12 to 5.39	1.13	−3.60 to 5.87	1.11	−3.60 to 5.87
At 60–64 years							
BMI, kg/m^2^							
Continuous		−0.07	−0.53 to 0.38	−0.10	−0.55 to 0.35	−0.11	−0.57 to 0.35
<18.5	4 (0.40)	9.83	−24.22 to 43.89	10.09	−23.8 to 44.04	9.07	−2.50 to 43.14
18.5–25	298 (29.68)	Reference		Reference		Reference	
25–30	419 (41.73)	−2.24	−7.37 to 2.87	−1.89	−7.00 to 3.22	−1.97	−7.11 to 3.18
≥30	283 (28.19)	−1.56	−7.17 to 4.05	−1.72	−7.31 to 3.88	−1.84	−7.53 to 3.84
Nonoverweight	302 (30.08)	Reference		Reference		Reference	
Overweight or obese	702 (69.92)	−2.10	−6.75 to 2.55	−1.95	−6.59 to 2.68	−2.05	−6.74 to 2.64
Waist circumference (cm)		−0.11	−0.28 to 0.06	−0.04	−0.22 to 0.14	−0.004	−0.22 to 0.14
Hip circumference (cm)		0.02	−2.01 to 0.24	−0.03	−0.26 to 0.20	−0.03	−0.27 to 0.19
Nonabdominally obese	493 (49.10)	Reference		Reference		Reference	
Abdominally obese	511 (50.90)	2.25	−2.01 to 6.52	1.19	−3.17 to 5.55	1.19	−3.17 to 5.55

*Note*: BMI = body mass index; LTL = leukocyte telomere length.

*Adjusted for sex.

^†^Additional adjustment for childhood SEP and adult SEP.

### Longitudinal Associations Between Adiposity and Telomere Length

Overall, there is an indication toward inverse associations between change in adiposity and telomere length (Table 3). An annual increase of one unit waist circumference between 53 and 60–64 was associated with a −0.60% greater annual decrease in LTL (95% CI: −1.31 to 0.10) which approached statistical significance (*p* = .09). This association was slightly weaker when adjusted for baseline waist circumference. Longitudinal associations between changes in BMI, weight or hip circumference and LTL were similar but weaker (Table 3).

**Table 3.** Associations Between Changes in BMI and Telomere Attrition

	ΔLTL*	
	Percent Difference	95% CI
Annual BMI change, kg/m^2^	−1.01	−3.14 to 1.13
Adjusted for baseline BMI	−0.93	−3.09 to 1.24
Annual weight change, kg	−0.25	−1.02 to 0.52
Adjusted for baseline weight	−0.26	−1.04 to 0.51
Annual waist circumference change, cm	−0.60	−1.31 to 0.10
Adjusted for baseline waist circumference	−0.59	−1.32 to 0.14
Annual hip circumference change, cm	−0.65	−1.57 to 0.26
Adjusted for baseline hip circumference	−0.69	−1.63 to 0.26

*Note:* All models were Adjusted for sex, childhood SEP, and adulthood SEP. Baseline measurements refer to measurements at age 53 years. BMI = body mass index; LTL = leukocyte telomere length.

*Annual percentage change in LTL.

### Associations Between BMI and Overweight in Earlier Life and Telomere Length

**Figure 1.**
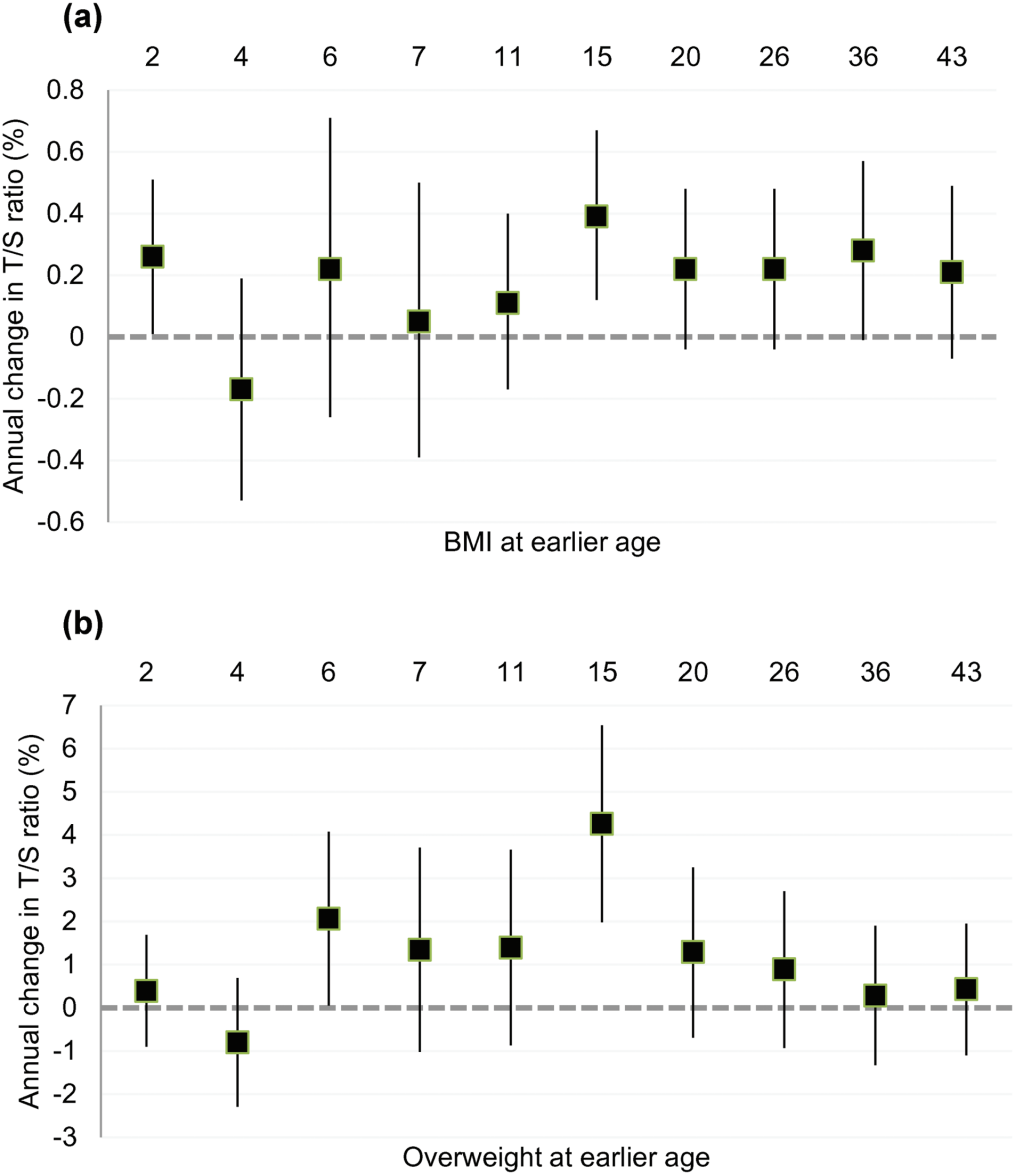
Associations between higher BMI (**a**) and overweight (**b**) and at earlier ages and changes in LTL (T/S ratio) between ages 53 and 60–64. For the latter (b), nonoverweight individuals were assigned as the reference. All models were adjusted for sex, childhood SEP, and adult SEP. BMI = body mass index; LTL = leukocyte telomere length; SEP = socioeconomic position.

Higher earlier BMI and overweight status were generally weakly associated with shorter LTL at age 53, but slightly longer LTL at 60–64 (Table 4). Higher BMI and overweight at previous ages therefore tended to be associated with less LTL attrition between ages 53 and 60–64 (Figure 1), most strongly for BMI and overweight at age 15 (eg, 0.39%, 95% CI: 0.12–0.67% annual change in LTL for each unit BMI increase at age 15).

**Table 4.** Associations of BMI at Earlier Ages or Overweight Status with Telomere Length and Attrition

Age of BMI Measurement	LTL at Age 53 Y*			LTL at Age 60–64 Y^†^			ΔLTL^‡^		
	*N* Overweight/ *N* Total	Percent Difference	95% CI	*N* Overweight/ *N* Total	Percent Difference	95% CI	*N* Overweight/ *N* Total	Percent Difference	95% CI
Continuous BMI									
2	690/1,972	−1.06	−2.07 to −0.05	812	0.51	−0.43 to 1.45	307/810	0.26	0.01 to 0.51
4	445/2,213	−0.25	−1.70 to 1.19	909	−0.19	−1.54 to 1.16	183/907	−0.17	−0.53 to 0.19
6	227/2,074	−1.04	−2.87 to 0.79	854	0.33	−1.47 to 2.12	96/852	0.22	−0.26 to 0.71
7	162/2,140	0.31	−1.41 to 2.04	887	0.53	−1.13 to 2.18	65/885	0.05	−0.39 to 0.50
11	177/2,156	0.40	−0.70 to 1.50	905	0.04	−1.01 to 1.08	72/903	0.11	−0.17 to 0.40
15	185/1,990	−0.54	−1.59 to 0.51	823	0.38	−0.60 to 1.38	78/822	0.39	0.12 to 0.67
20	241/2,034	−0.36	−1.44 to 0.72	857	0.05	−0.93 to 1.02	109/855	0.22	−0.04 to 0.48
26	444/2,201	−0.35	−1.37 to 0.67	929	0.57	−0.33 to 1.46	170/927	0.22	−0.04 to 0.48
36	775/2,262	−0.68	−1.73 to 0.37	950	0.44	−0.52 to 1.40	309/948	0.28	−0.01 to 0.57
43	1,101/2,335	−0.77	−1.82 to 0.27	964	0.36	−0.53 to 1.26	434/962	0.21	−0.07 to 0.49
Overweight									
2	690/1,972	−2.64	−7.84 to 2.56	308 (37.93)	0.24	−4.68 to 5.15	307/810	0.39	−0.90 to 1.69
4	445/2,213	0.91	−4.94 to 6.77	184 (20.24)	0.62	−4.50 to 6.20	183/907	−0.80	−2.29 to 0.69
6	227/2,074	−5.40	−13.17 to 2.37	96 (11.24)	4.63	−2.72 to 11.99	96/852	2.06	0.05 to 4.08
7	162/2,140	−0.67	−9.78 to 8.45	65 (7.33)	7.24	−1.52 to 16.00	65/885	1.34	−1.02 to 3.71
11	177/2,156	−2.76	−11.66 to 6.14	72 (7.96)	−1.46	−9.85 to 6.93	72/903	1.39	−0.87 to 3.66
15	185/1,990	−7.00	−15.97 to 1.97	78 (9.48)	5.16	−2.94 to 13.27	78/822	4.26	1.98 to 6.54
20	241/2,034	−3.84	−12.08 to 4.39	109 (12.72)	−1.56	−8.82 to 5.51	109/855	1.29	−0.69 to 3.25
26	444/2,201	−0.65	−7.43 to 6.12	170 (18.30)	−1.53	−8.01 to 4.95	170/927	0.89	−0.93 to 2.70
36	775/2,262	1.70	−4.62 to 8.03	309 (32.53)	−0.75	−5.61 to 4.10	309/948	0.28	−1.33 to 1.90
43	1,101/2,335	0.45	−5.66 to 6.56	435 (45.12)	0.73	−3.74 to 5.20	434/962	0.43	−1.10 to 1.95

*Note*: All models are adjusted for sex, childhood SEP, and adult SEP. For analysis of overweight status, nonoverweight individuals were assigned as the reference. BMI = body mass index; LTL = leukocyte telomere length; SEP = socioeconomic position.

*Adjusted for BMI at age 53 years.

^†^Adjusted for BMI at age 60–64 years.

^‡^Annual percentage change in LTL. Adjusted for baseline BMI at age 53 years.

## Discussion

We observed a weak inverse association between gain in adiposity and telomere attrition over the same time period. There was evidence of associations of earlier life BMI and LTL attrition, with higher BMI and overweight at 15 years being associated with less LTL attrition in midlife.

Common biological pathways linking obesity and ageing outcomes have been identified, which support our findings on concurrent BMI gain and telomere attrition in midlife. Increasing adiposity may induce oxidative stress (22), which have been linked to reduced activity of telomerase (23), the enzyme responsible for telomere maintenance. In addition to affecting telomerase pathways, adipose tissue in obese animal models shows signs of accumulating DNA damage and cellular ageing such as higher levels of p53 protein (24). An indirect role of obesity may also occur via circulating glucose and lipids, commonly elevated in overweight or obese individuals. High circulating glucose increases the generation of advanced glycated end products (25), and high lipid levels may induce lipid peroxidation (26), both of which contribute to oxidative stress and inflammation. Additionally, hyperinsulinemia which often accompanies obesity stimulates the mechanistic target of rapamycin (mTOR), whose blockade results in prolonged lifespan in mice (27). A recent study suggested a beneficial effect of mTOR by mediating the anti-obesity effects of fibroblast growth factor 21 (FGF21) (28), implying complex pathways linking obesity and ageing which may involve negative feedback mechanisms.

We did not find cross-sectional associations between BMI or overweight status and telomere length in midlife, which is similar to a lack of association observed in a meta-analysis (10). Nevertheless, point estimates at both ages 53 and 60–64 suggested a weak inverse trend in the fully adjusted models, corroborating shorter telomeres with higher adiposity that we recently reported in American populations (29). Longitudinally, the weak association between greater adiposity gain and greater telomere attrition in midlife supports the inverse correlation between 5-year changes in adiposity and telomere length in the PREDIMED-NAVARRA trial (12). With respect to waist circumference, each *SD* change within a 6-year observation was reported to longitudinally correspond to 41.84 base pair LTL decrease (*SE*: 16.39 base pair) in a study including 2,981 Dutch individuals aged 18–65 (30). On the other hand, a Danish study including over 4,500 adult Caucasians showed a lack of association between weight change and telomere attrition (31). This may indicate that weight change correlates to a different extent with telomere length than fat distribution. Although variation in telomere assays has been reported to hamper comparability (32,33), all aforementioned studies used rt-PCR to assess relative telomere length, suggesting additional factors, such as age- and cohort-specific characteristics, to contribute to this discrepancy.

Our findings suggested that there was little consistent association between early life BMI and LTL in midlife. Positive associations were seen between early life adiposity, particularly at age 15, and LTL change between ages 53 and 60–64. With regards to LTL at younger ages, a study based on the Northern Finland Birth Cohort 1966 observed a positive association between BMI measured at childhood adiposity rebound (age ~5 years) with LTL at age 31 in women (34). Also in the same study, less telomere attrition between ages 60 and 70 was seen with greater gain in BMI between ages 12 and 24 months (34). A German cohort of 1,000 adults aged 50–75 also saw nonsignificant trends suggesting less 8-year telomere attrition with longer duration of overweight since age 20. Together, these findings point towards a possible benefit of having larger body size earlier in life on telomere length in mid- to late adulthood. We speculate that this phenomenon may support the “thrifty telomere” hypothesis (35), which proposes that telomere length acts as a marker of one’s limited resources over the life course, known as “disposable soma” (36). This theory suggests having shorter telomeres to start with results in a more thrifty investment in maintenance efforts and reduced cell proliferation (35). Since telomere shortens with each cell replication (5), this eventually leads to decreased telomere attrition. Such a mechanism is suggested to explain less telomere attrition with shorter LTL at baseline as seen in our study and others (37,38).

The strength on this study lies in the repeated measurements of BMI since childhood through adulthood, and two repeated assessments of adiposity and LTL at the same ages in midlife which allowed a longitudinal analysis. We used a measure of relative telomere length which showed better comparability across laboratories than absolute measures of telomere length (33). A limitation of this study is that misclassification may have occurred when classifying individuals based on BMI, waist, and hip circumference since they do not directly reflect body composition. However, BMI and waist circumference has been shown to be equally relevant for cardiovascular risk factors in comparison with fat mass (39). Individuals who had higher BMI were at higher risk of cardiovascular disease and may die prior to LTL assessment at age 53, and this may result in underestimation of the observed associations.

In summary, we found little evidence on longitudinal association between adiposity and telomere length and attrition in midlife. Additionally, we found a positive association between early life overweight and telomere attrition in midlife which may support the “thrifty telomere” hypothesis and indicate that being overweight in early life could alter telomere maintenance in later life. Further investigations are needed to assess whether telomere attrition lies in the pathways between early life adiposity and ageing-related outcomes.

## Funding

This work was supported by the UK Medical Research Council which provides core funding for the MRC National Survey of Health and Development and R.H. (MC_UU_12019/2) and D.K. (MC_UU_12019/1 and 12019/4).

## Conflict of interest

None declared.
